# Supplementary material for: The role of experience in prioritizing hippocampal replay
Source: Nat Commun. 2023 Dec 9;14:8157. doi: 10.1038/s41467-023-43939-z (PMC10710481; doi:10.1038/s41467-023-43939-z)
Supplement: Supplementary file 1 — Supplementary Information [file 41467_2023_43939_MOESM1_ESM.pdf]

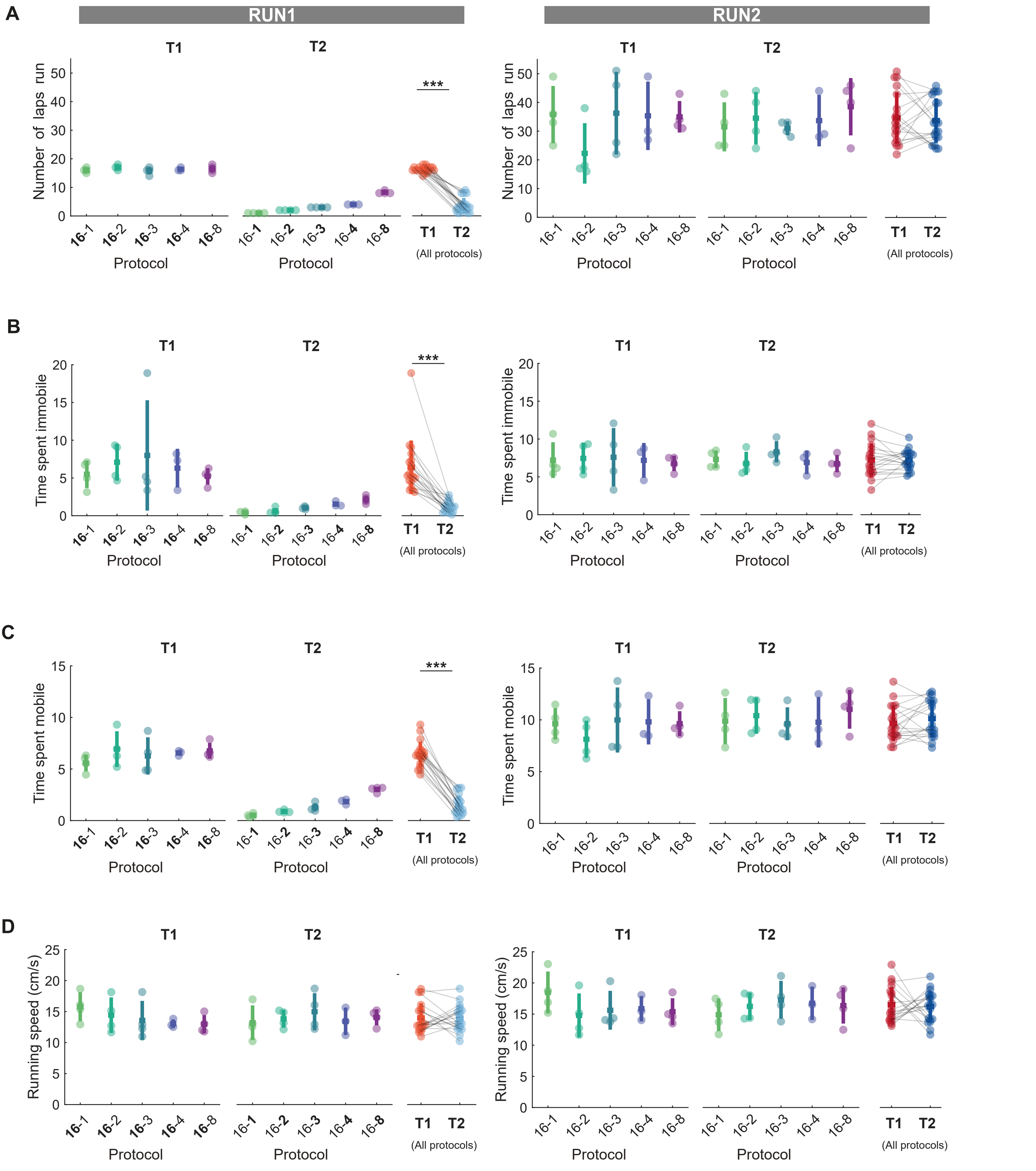

Figure S1. Summary of animal behavior during RUN1 and RUN2 for each protocol. (A-D) The behavioral metrics on track 1 and track 2 during RUN1 (left) and RUN2 (right) for each protocol. The lap number associated with each track during RUN1 was made in bold. The lap number in RUN2 was not highlighted as the time spent on two tracks were nearly identical. Each data point represents one of the four rats tested for a given track within a session, color-coded according to the experimental protocol and track identity. (***p<0.001, two-tailed Wilcoxon signed rank test). **(A)** Number of laps run for each protocol. ***p = 0.00013 for T1 Vs T2 RUN1 and p = 0.72 for T1 Vs T2 RUN2. **(B)** Time spent immobile for each protocol. ***p = 0.00013 for T1 Vs T2 RUN1 and p = 0.78 for T1 Vs T2 RUN2. **(C)** Time spent mobile for each protocol. ***p = 0.00013 forT1 Vs T2 RUN1 and p = 0.26 for T1 Vs T2 RUN2. **(D)** Moving speed for each protocol. p = 0.94 for T1 Vs T2 RUN1 and p = 0.97 for T1 Vs T2 RUN2. n = 19 sessions from 4 rats. Data point from Track 1 (T1) and Track 2 (T2) during first exposure (RUN1) are indicated using orange and light blue and Track 1 and Track 2 during second exposure (RUN2) are indicated using red and dark blue. Error bars are presented as mean ± SD for panels **(A-D).**

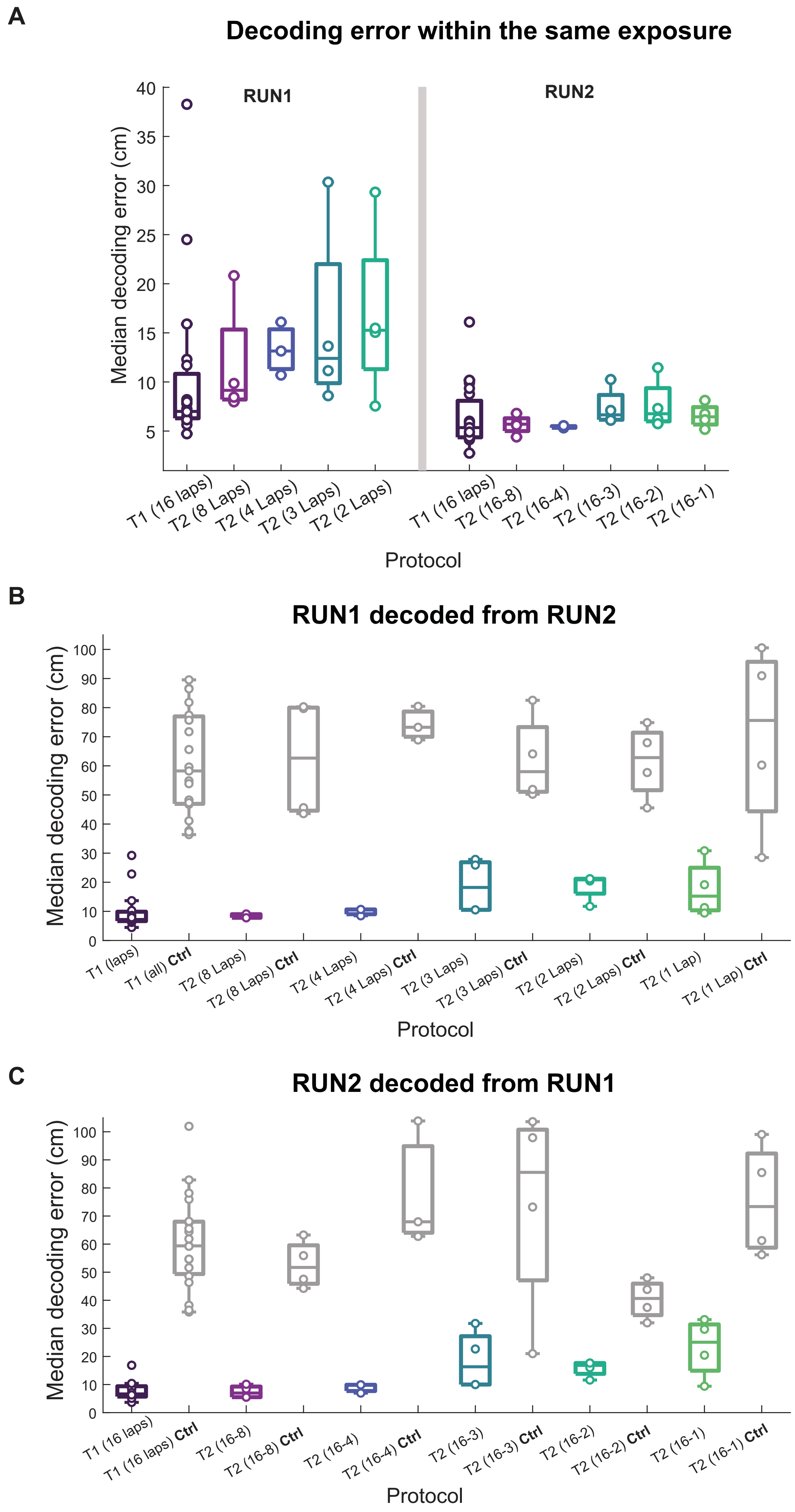

Figure S2. Summary of median decoding errors across protocols and tracks (A) Median decoding error within an exposure based on place fields from the final lap to decode the preceding laps (RUN1 T2) or laps 13-16 to decode laps 1-12 (RUN1 T1 and RUN2 T1+T2), **(B)** RUN1 decoded from RUN2 place fields. **(C)** RUN2 decoded from RUN1 place fields. *Ctrl* (in **B,C**) indicates a control analysis where place fields from the opposite track are used for decoding. Each data point represents median decoding error from a session, color-coded according to the experimental protocol (same as Figure S1). n = 19 sessions from 4 rats.

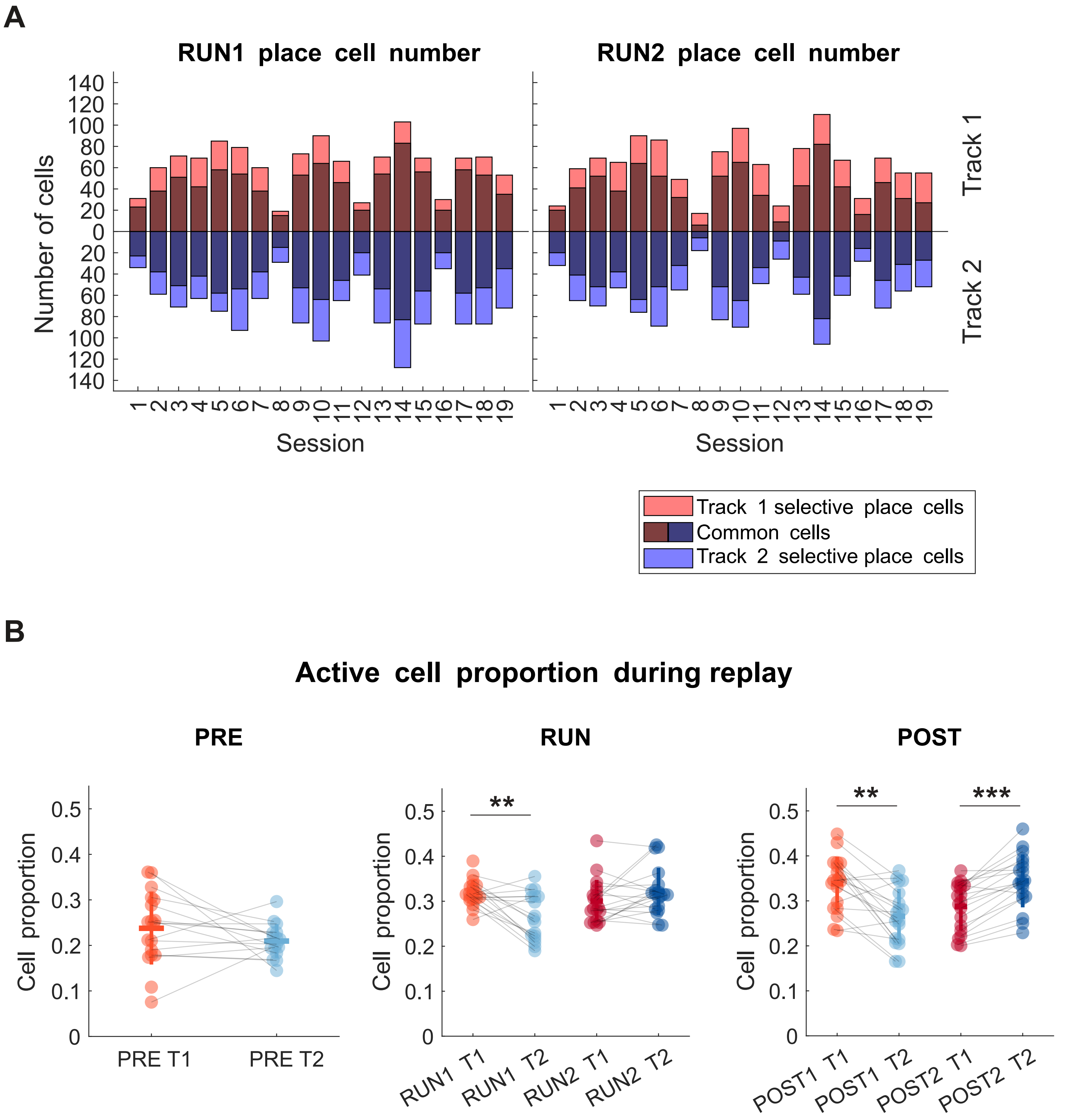

Figure S3. Number of place cells and place cell participation across experiments (A) Summary of number of place cells in each recording session for RUN1 (left) and RUN2 (right). Place cells were either selective for track 1 (light red), selective for track 2 (light blue) or had place fields on both tracks (dark red/blue). (**B**) Proportion of active cells during sleep replay events for PRE (left), RUN 1+2 (center), and POST 1+2 (right). p = 0.11 for T1 vs T2 PRE replay, **p = 0.0075 for T1 vs T2 RUN1 replay, p = 0.14 for T1 vs T2 RUN2 replay, **p = 0.0011 for T1 vs T2 POST1 replay, ***p = 0.00018 for T1 vs T2 POST2 replay, two-tailed Wilcoxon signed rank test. n = 19 sessions from 4 rats. Error bars are presented as mean ± SD. Each data point is color-coded according to the experimental protocol and track identity (same as Figure S1).

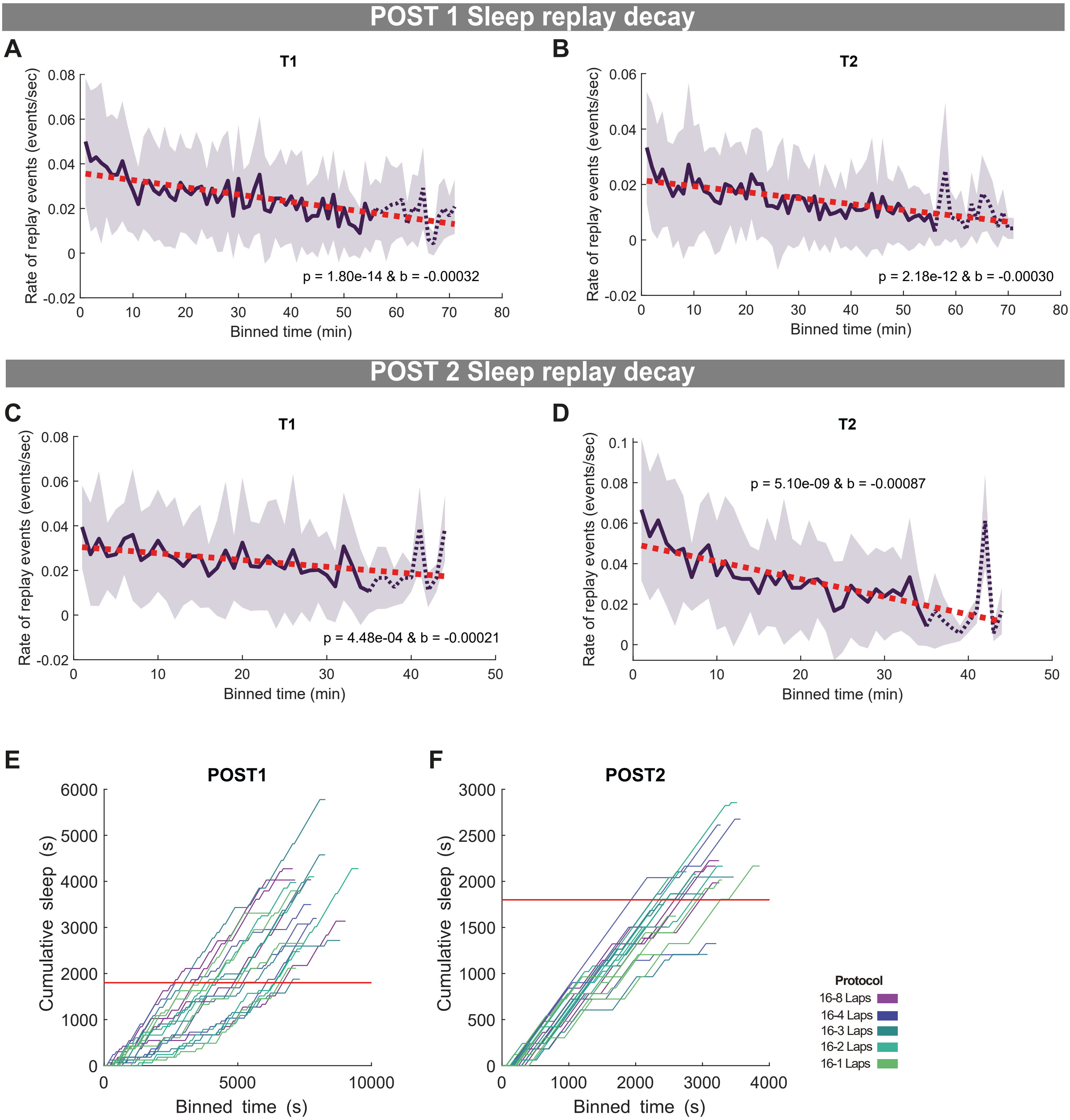

Figure S4. Decay of sleep replay rate for track 1 and track 2 events during POST1 and POST2. (A-D) Regression between the binned cumulative sleep time and the rate of sleep replay for **(A)** track 1 and **(B)** track 2 events during POST1 and **(C)** track 1 and **(D)** track 2 events during POST2. The solid line and shaded region represent the mean and standard deviation across all sessions for a given protocol. The solid line becomes dashed line when less than half of the animals are contributing to the data at each time point. (**E-F**) Cumulative sleep across time for each experiment for (**E**) POST1 and (**F**) POST2. Each line represents sleep data from a session, which color-coded according to the experimental protocol (same as Figure S1). n = 19 sessions from 4 rats.

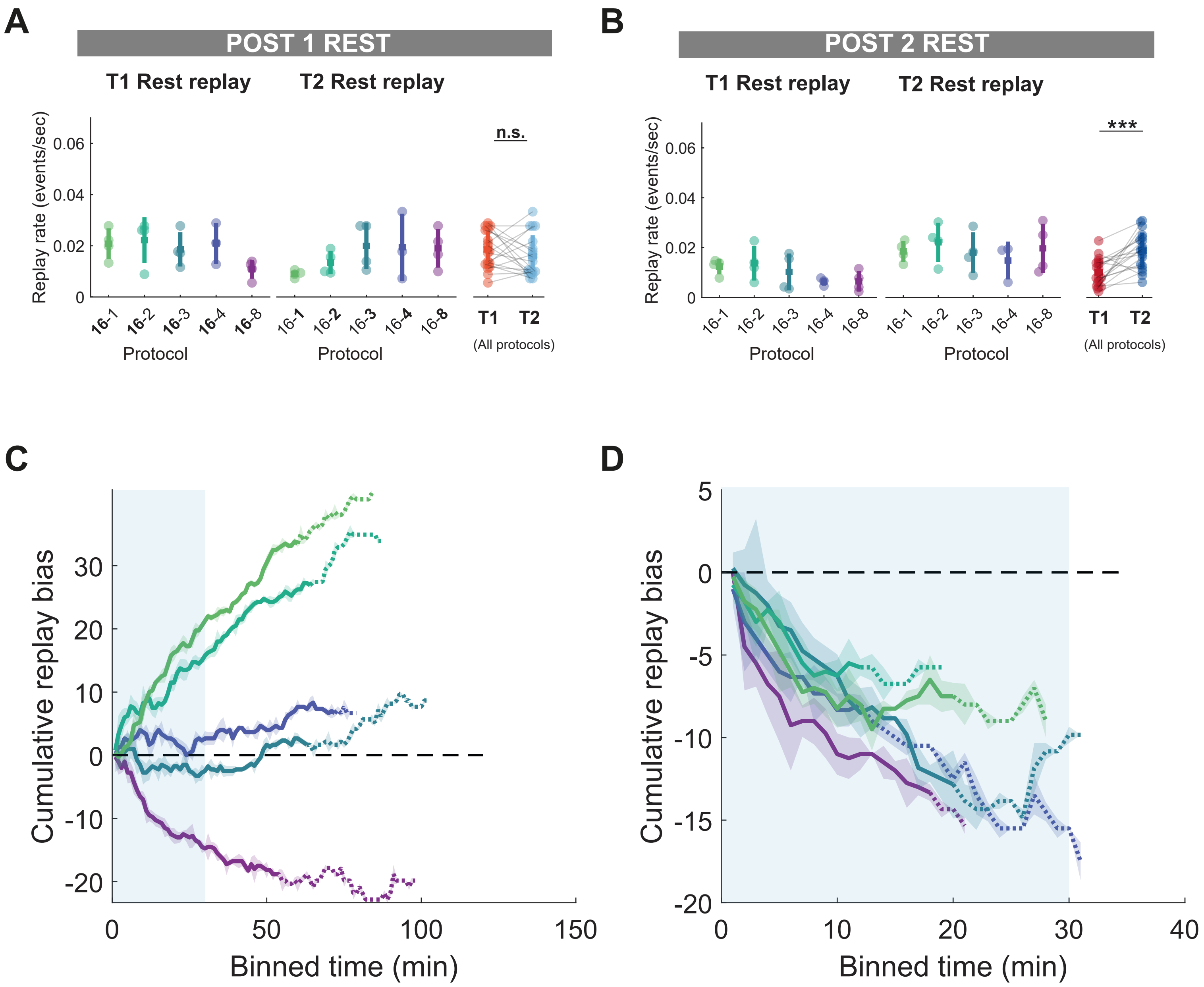

Figure S5. Hippocampal POST rest replay is sensitive to contextual novelty and familiarity. (A,B) Rate of rest replay for track 1 and track 2 during first 30 mins of cumulative sleep of POST1 **(A)** and POST2 **(B)**.). For POST1, the lap number associated with each track during RUN1 was made in bold. For POST2, the lap number was not highlighted as the time spent on two tracks were nearly identical. Each data point represents the mean rest replay rate within a session, color-coded according to the experimental protocol (same as Figure S1). $p = 0.32$ for T1 Vs T2 rest POST1 replay,***$p = 0.0010$ for T1 Vs T2 rest POST2 replay, two-tailed Wilcoxon signed rank test). $n = 19$ sessions from 4 rats. Error bars are presented as mean ± SD. **(C,D)** Cumulative rest replay bias across protocols during POST1 **(C)** and POST2 **(D)**. The solid line and shaded region represents the mean and standard deviation across all sessions for a given protocol. The solid line becomes dashed line when less than half of the animals are contributing to the data at each time point. Each line is color-coded according to the experimental protocol (same as Figure S1). The light blue box outlined the first 30 mins of cumulative rest time windows used for analysis in **(A,B).**

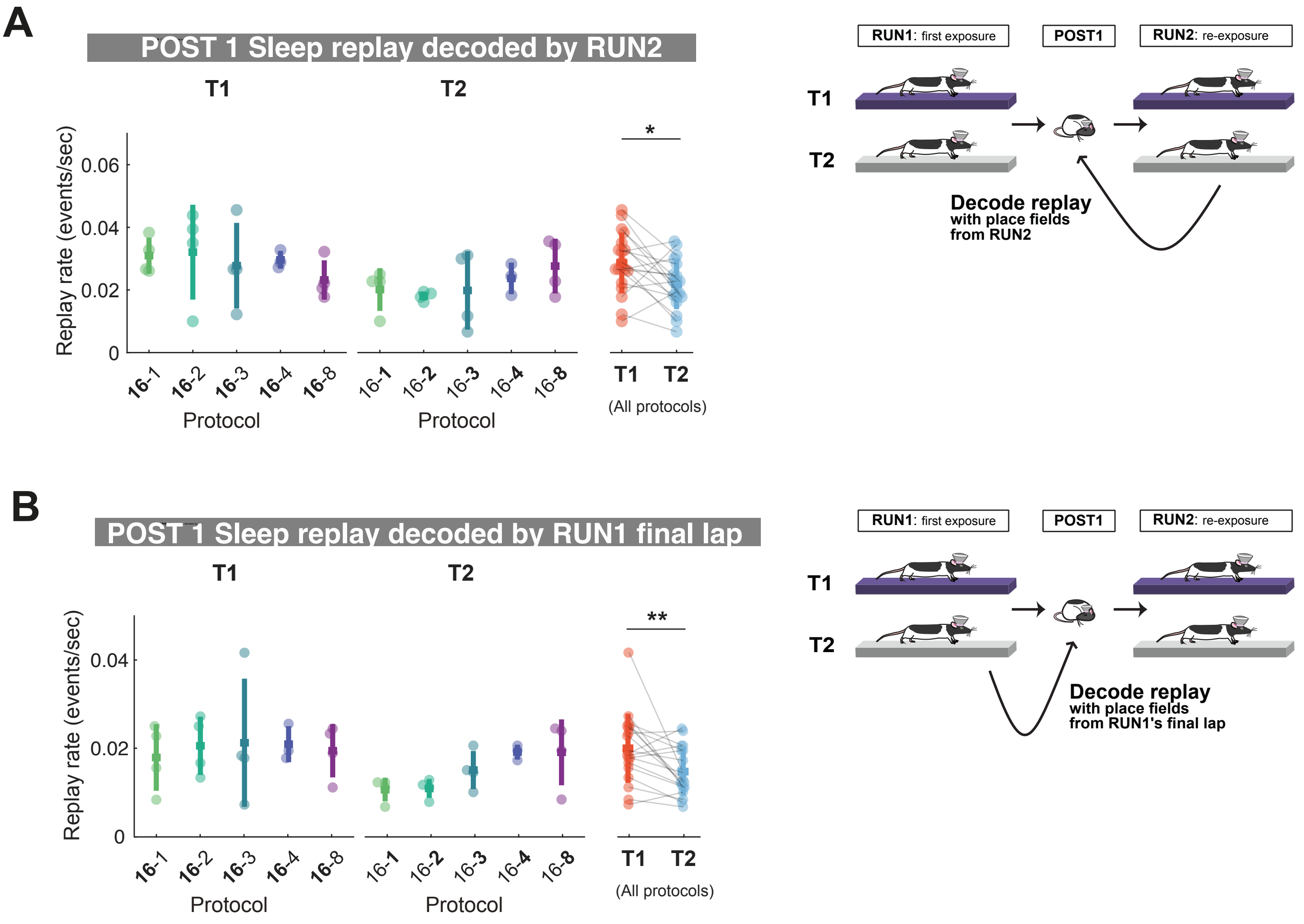

Figure S6. Sleep replay rate track difference during POST1 remained significantly different when alternative place field templates were used for replay decoding. Rate of sleep replay for track 1 and track 2 during first 30 mins of cumulative sleep of POST1, after detecting replay events decoded using place fields from **(A)** RUN2 and **(B)** the final lap from RUN1. The lap number associated with each track during RUN1 was made in bold. Each data point represents the mean sleep replay rate within a session, color-coded according to the experimental protocol (same as Figure S1). *p = 0.018 **(A)**, **p = 0.0070 **(B)**, two-tailed Wilcoxon signed rank test. n = 19 sessions from 4 rats. Error bars are presented as mean ± SD.

Awake replay and theta sequence VS POST rest replay

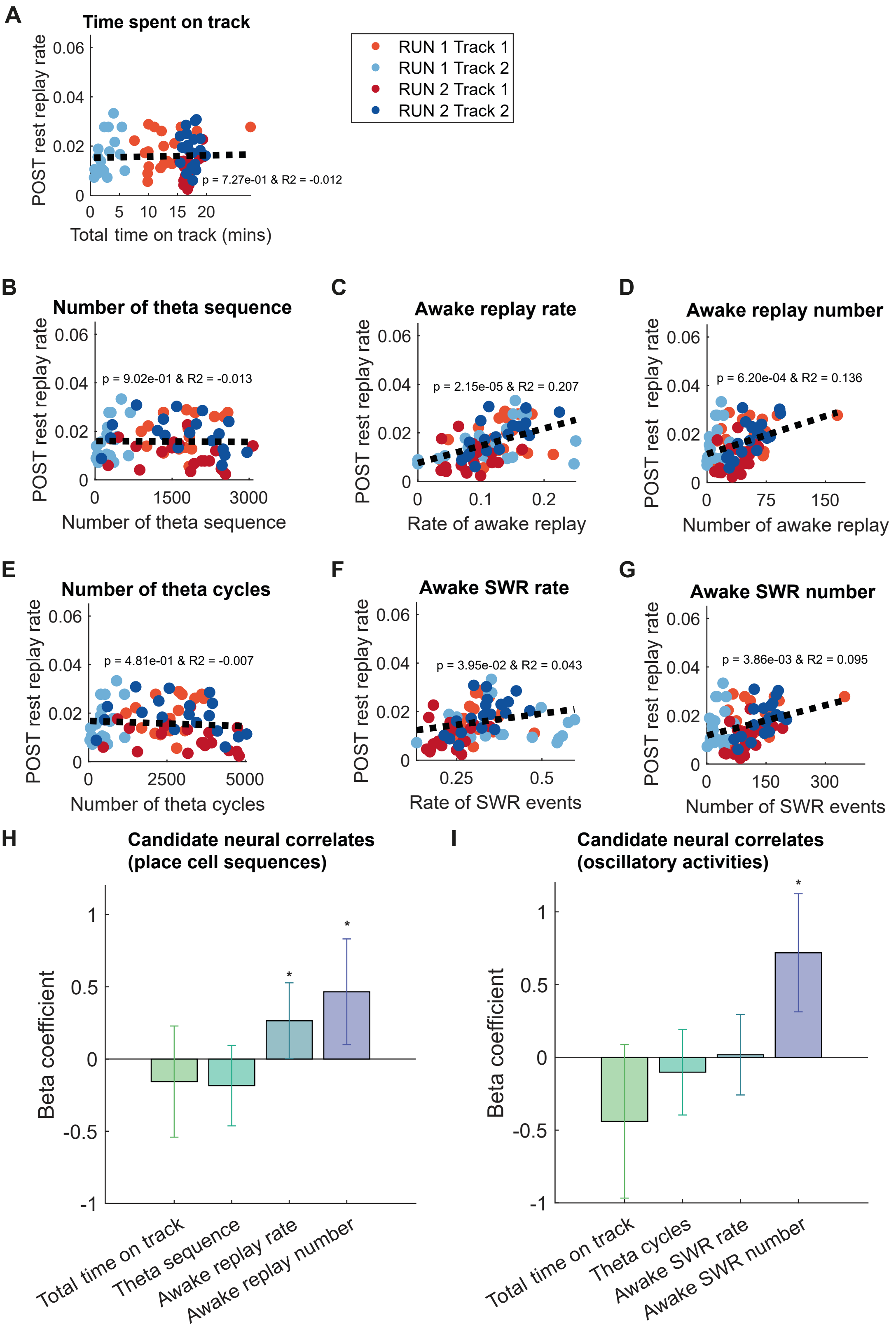

Figure S7. The predictive relationship between the awake replay and theta sequence and POST rest replay. (A-G) Simple linear regression of behavioral or neural metric and rate of POST rest replay during first 30 mins of cumulative sleep. **(A)** Time spent on track. **(B)** Number of theta sequence. **(C)** Awake replay rate. **(D)** Awake replay number. **(E)** Number of theta cycles **(F)** Awake SWR rate **(G)** Awake SWR number. Each data point is color-coded according to the experimental protocol and track identity (same as Figure S1). n = 76 data points from both tracks during RUN1 and RUN2 of 19 sessions (4 rats). **(H-I)** Mixed-effect regression for the relationship between candidate neural correlates and rest replay: **(H)** Place cell sequences **(I)** Oscillatory events. Each bar indicates the magnitude of the standardized beta coefficient associated with different factors with the error bar showing the estimated 95% confidence interval. Asterisks (*) where the 95% confidence interval of the standardized beta coefficient does not overlap with 0.

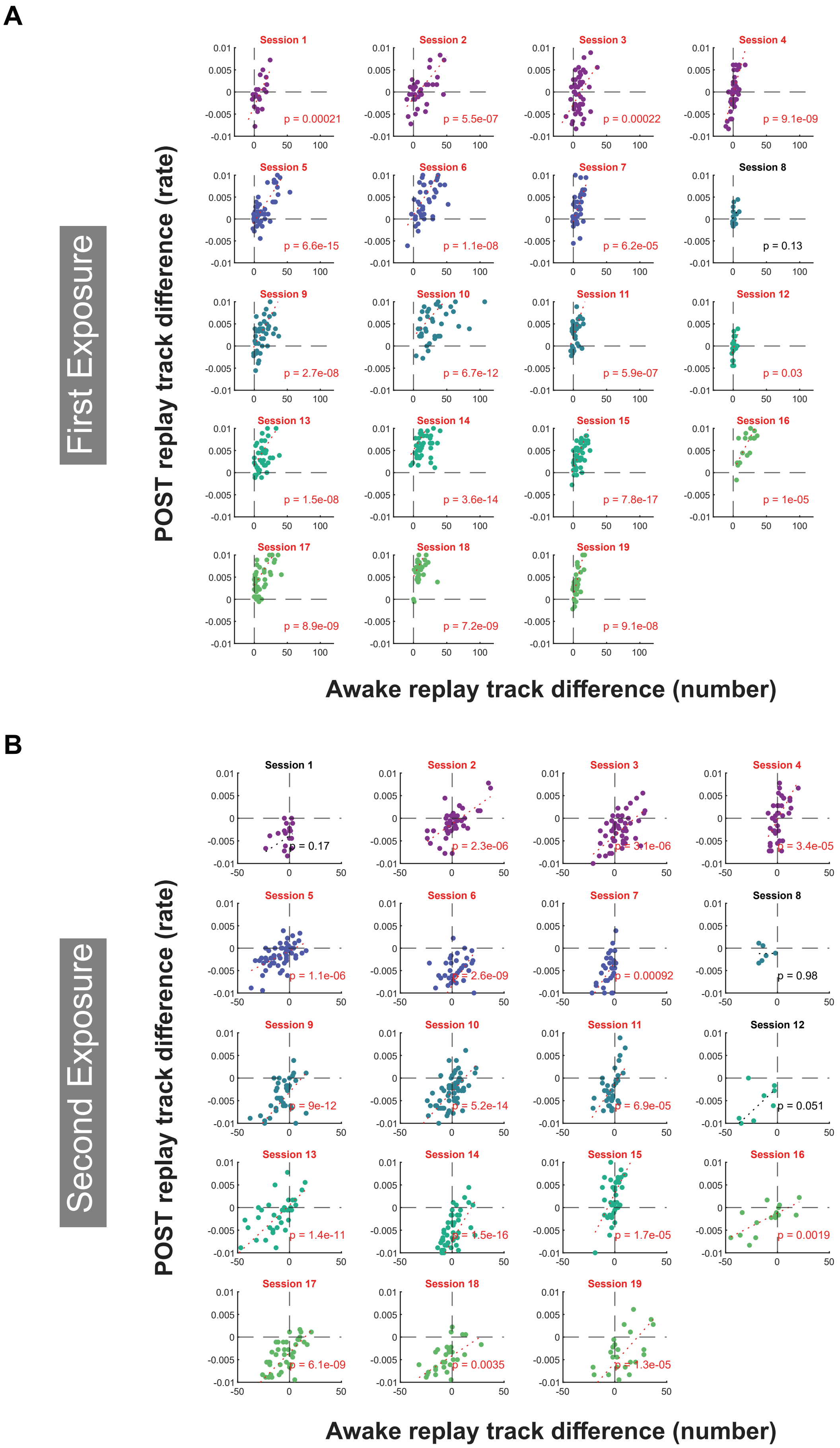

Figure S8: Place cells participate in more sleep replay events if they participate in more local awake replay events during RUN. (A,B) Regression between awake replay track difference (number) and POST sleep replay track difference (rate) for first exposure and second exposure. Each data point is a place cell with place fields on both tracks for a given session. For each place cell, the difference in the number of local awake replay events (track 1 - track 2) a given cell was active during RUN was regressed with the observed difference in sleep replay rates (track 1 - track 2) for that cell during the subsequent POST1 **(A)** or POST2 **(B).** Each subplot contains data points (place cells) from one session, color-coded according to the experimental protocol (same as Figure S1). Sessions with a statistically significant regression ($p < 0.05$) are highlighted in red. See source data associated with this figure to see the number of data points (number of place cells with place fields on both tracks) for each session.

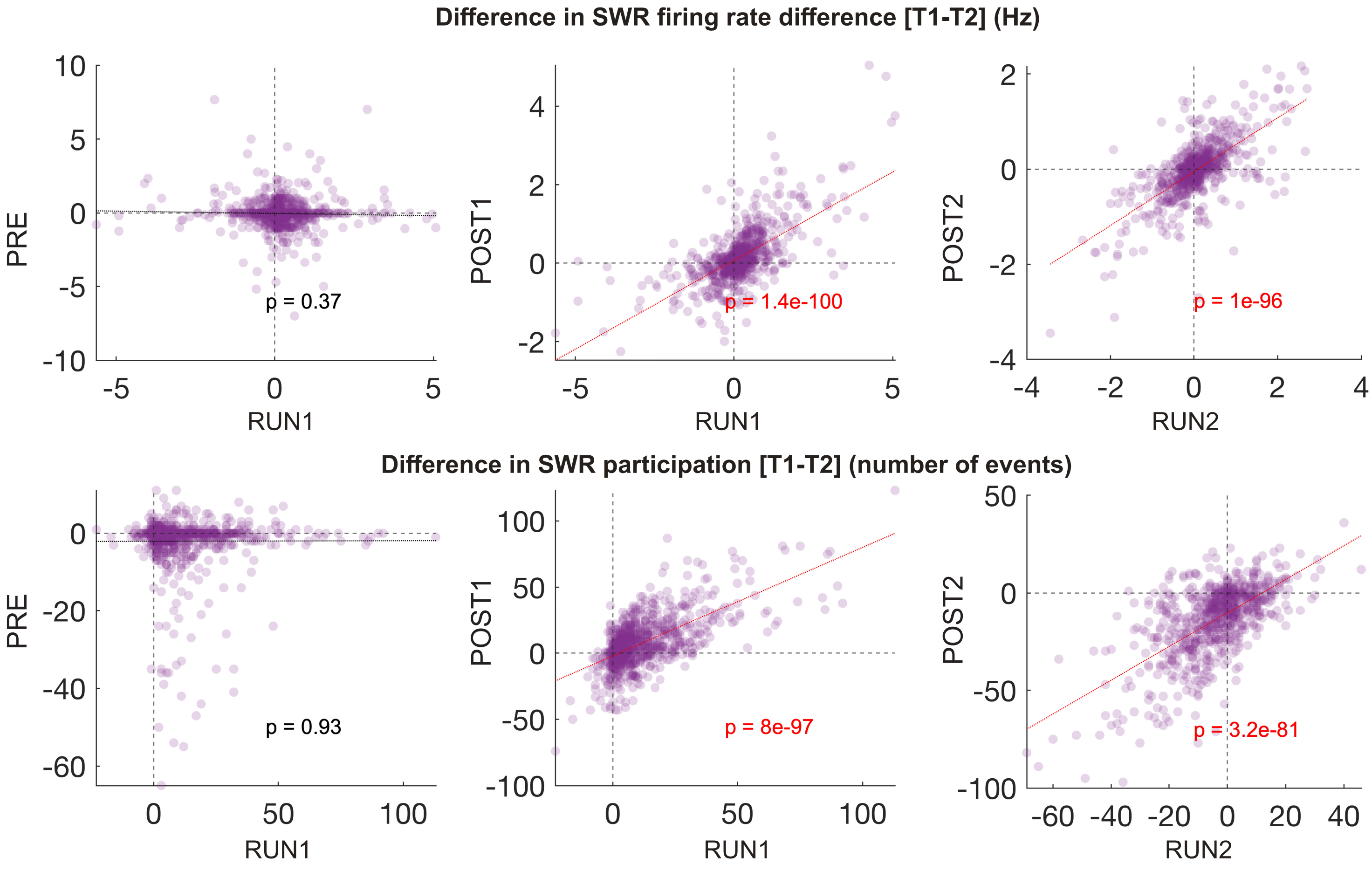

Figure S9. Place cell SWR participation track difference during PRE, RUN and POST. Simple linear regression of difference in SWR firing rate (top) and SWR event participation (bottom) for PRE vs RUN1 (left), POST1 vs RUN1 (centre), and POST2 vs RUN2 (right). A statistically significant regression ($p < 0.05$) is highlighted in red. n = 807 place cells with place fields on both tracks from 19 sessions for RUN1 VS PRE and RUN1 VS POST. n = 709 place cells with place fields on both tracks from 19 sessions for RUN2 vs POST2.

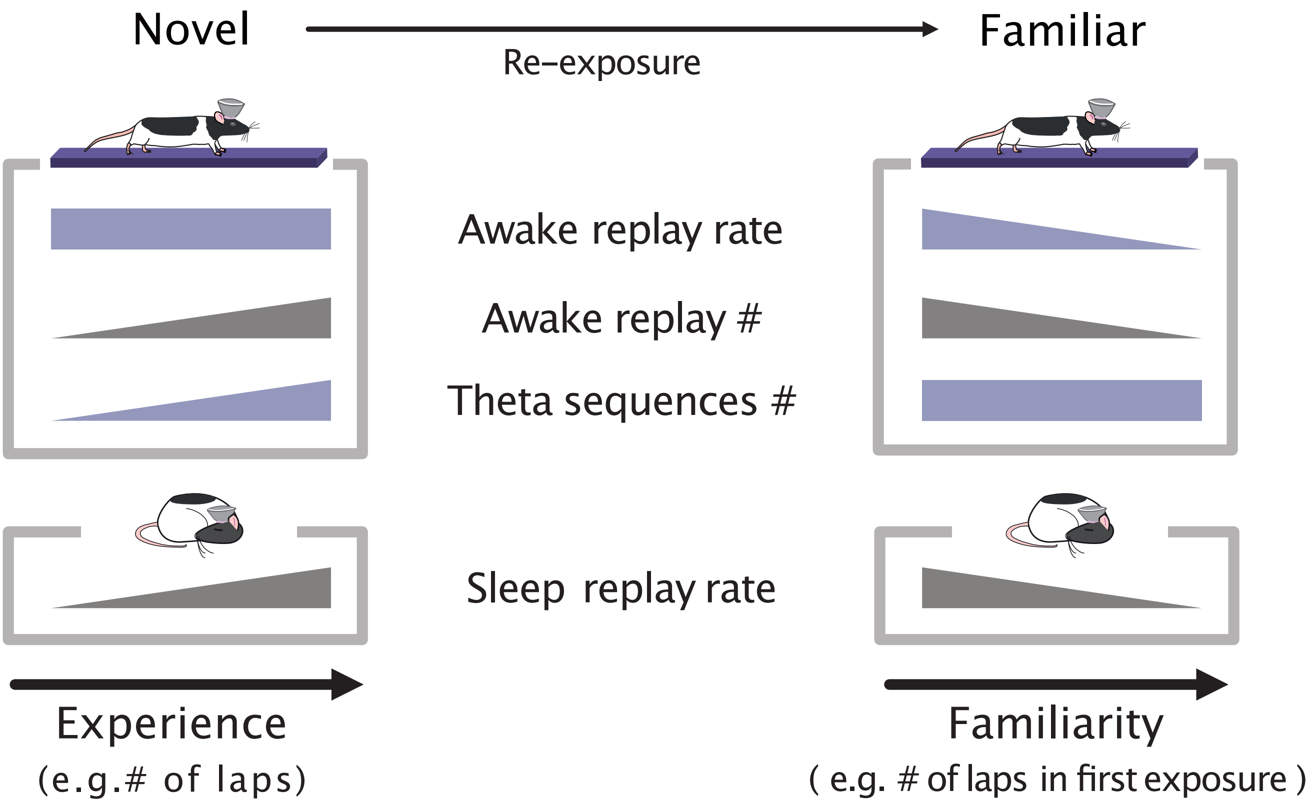

Figure S10. Summary schematics of the role of experience in prioritizing hippocampal replay.
