# Supplementary material for: The role of experience in prioritizing hippocampal replay
Source: Nat Commun. 2023 Dec 9;14:8157. doi: 10.1038/s41467-023-43939-z (PMC10710481; doi:10.1038/s41467-023-43939-z)
Supplement: Supplementary file 3 — Reporting Summary [file 41467_2023_43939_MOESM3_ESM.pdf]

nature portfolio

Corresponding author(s): Daniel Bendor

Last updated by author(s): Nov 8, 2023

Reporting Summary

Nature Portfolio wishes to improve the reproducibility of the work that we publish. This form provides structure for consistency and transparency in reporting. For further information on Nature Portfolio policies, see our Editorial Policies and the Editorial Policy Checklist.

Statistics

For all statistical analyses, confirm that the following items are present in the figure legend, table legend, main text, or Methods section.

n/a	Confirmed	
☐	☒	The exact sample size (*n*) for each experimental group/condition, given as a discrete number and unit of measurement
☐	☒	A statement on whether measurements were taken from distinct samples or whether the same sample was measured repeatedly
☐	☒	The statistical test(s) used AND whether they are one- or two-sided<br>*Only common tests should be described solely by name; describe more complex techniques in the Methods section.*
☐	☒	A description of all covariates tested
☐	☒	A description of any assumptions or corrections, such as tests of normality and adjustment for multiple comparisons
☐	☒	A full description of the statistical parameters including central tendency (e.g. means) or other basic estimates (e.g. regression coefficient) AND variation (e.g. standard deviation) or associated estimates of uncertainty (e.g. confidence intervals)
☐	☒	For null hypothesis testing, the test statistic (e.g. *F*, *t*, *r*) with confidence intervals, effect sizes, degrees of freedom and *P* value noted<br>*Give P values as exact values whenever suitable.*
☒	☐	For Bayesian analysis, information on the choice of priors and Markov chain Monte Carlo settings
☒	☐	For hierarchical and complex designs, identification of the appropriate level for tests and full reporting of outcomes
☐	☒	Estimates of effect sizes (e.g. Cohen's *d*, Pearson's *r*), indicating how they were calculated

Our web collection on statistics for biologists contains articles on many of the points above.

Software and code

Policy information about availability of computer code

Data collection	Data were collected using Neuralynx Cheetah acquisition software
Data analysis	Data analysis was performed using custom scripts in MATLAB R2017-R2021a (avaliable on Github https://github.com/bendor-lab/Nat_Com_Huelin_Gorriz_et_al).

For manuscripts utilizing custom algorithms or software that are central to the research but not yet described in published literature, software must be made available to editors and reviewers. We strongly encourage code deposition in a community repository (e.g. GitHub). See the Nature Portfolio guidelines for submitting code & software for further information.

Data

Policy information about availability of data

All manuscripts must include a data availability statement. This statement should provide the following information, where applicable:

- Accession codes, unique identifiers, or web links for publicly available datasets
- A description of any restrictions on data availability
- For clinical datasets or third party data, please ensure that the statement adheres to our policy

All data reported in this study are available from the corresponding authors upon request. Currently, the dataset is subject to ongoing analysis in several follow-up studies. We intend to make the data publicly available in a public repository upon the completion of our analyses.

April 2023

Research involving human participants, their data, or biological material

Policy information about studies with human participants or human data. See also policy information about sex, gender (identity/presentation), and sexual orientation and race, ethnicity and racism.

Reporting on sex and gender	N/A
Reporting on race, ethnicity, or other socially relevant groupings	N/A
Population characteristics	N/A
Recruitment	N/A
Ethics oversight	N/A

Note that full information on the approval of the study protocol must also be provided in the manuscript.

Field-specific reporting

Please select the one below that is the best fit for your research. If you are not sure, read the appropriate sections before making your selection.

☒ Life sciences ☐ Behavioural & social sciences ☐ Ecological, evolutionary & environmental sciences

For a reference copy of the document with all sections, see nature.com/documents/nr-reporting-summary-flat.pdf

Life sciences study design

All studies must disclose on these points even when the disclosure is negative.

Sample size	Sample size was determined according to the convention in the hippocampal field (which range from 3 rats (Diba and Buzsaki 2007) to 6 rats (Shin et al 2019)). We recorded 19 sessions from 4 animals, which is comparable to some studies such as Foster and Wilson (2006) that used 4 rats. For each session, we recorded sufficient number of neurons for decoding of spatial location.
Data exclusions	One session out of 20 sessions were excluded due to low cell yield and poor spatial decoding.
Replication	All key results were consistent across 4 animals and 19 sessions. We did not use a separate group of animals to test for replicability.
Randomization	For RUN1 Track 2 running, the number of laps run on the track changed every day and the order was pseudo-randomized for each animal. The track configuration was also pseudo-randomized.
Blinding	During data collection, the experimenters were not blinded to the experimental conditions as they involved in setting up the track configuration and moving animals on and off the tracks. However, all analyses of both neural and behavioral data were automated and identical for all experimental conditions.

Reporting for specific materials, systems and methods

We require information from authors about some types of materials, experimental systems and methods used in many studies. Here, indicate whether each material, system or method listed is relevant to your study. If you are not sure if a list item applies to your research, read the appropriate section before selecting a response.

Materials & experimental systems

n/a	Involved in the study	
☒	☐	Antibodies
☒	☐	Eukaryotic cell lines
☒	☐	Palaeontology and archaeology
☐	☒	Animals and other organisms
☒	☐	Clinical data
☒	☐	Dual use research of concern
☒	☐	Plants

Methods

n/a	Involved in the study	
☒	☐	ChIP-seq
☒	☐	Flow cytometry
☒	☐	MRI-based neuroimaging

Animals and other research organisms

Policy information about studies involving animals; ARRIVE guidelines recommended for reporting animal research, and Sex and Gender in Research

Laboratory animals	4 male adult Lister Hooded rats with age ranging from 3 to 6 months old.
Wild animals	No wild animals were used in this study
Reporting on sex	All rats used for this study were male. As per home office license requirement, we need to achieve a minimum hyperdrive/body weight ratio for each animal. This is more realistically satisfied in male rats, given that they are larger and heavier. Furthermore, we are not aware of any previous rodent replay studies that balance the sex of rats.
Field-collected samples	No field collected samples were used in the study
Ethics oversight	All experimental procedures and post-operative care were approved and carried out in accordance with the UK Home Office, subject to the restrictions and provisions contained in the Animals (Scientific Procedures) Act of 1986.

Note that full information on the approval of the study protocol must also be provided in the manuscript.

Plants

Seed stocks	N/A
Novel plant genotypes	N/A
Authentication	N/A
